# Supplementary material for: The interactome of intact mitochondria by cross-linking mass spectrometry provides evidence for coexisting respiratory supercomplexes
Source: Mol Cell Proteomics. 2017 Dec 8;17(2):216–32. doi: 10.1074/mcp.RA117.000470 (PMC5795388; doi:10.1074/mcp.RA117.000470)
Supplement: Supplemental Data [file supp_RA117.000470_133922_0_supp_23978_fzffwf.zip › spectra_annotation/mito_spectra_annotation/483-1-1-1-8-1.pdf]

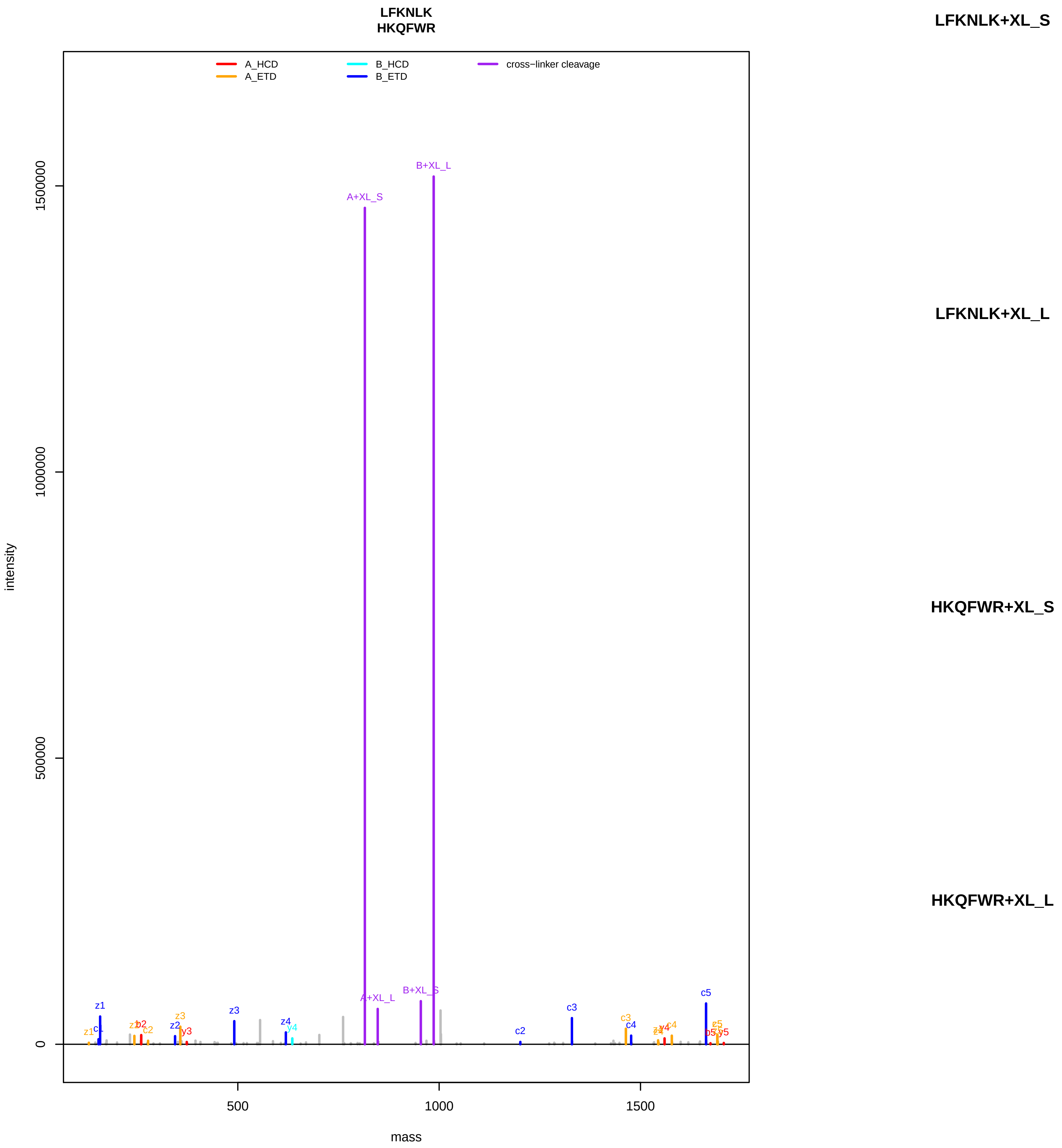

LFKNLK
HKQFWR
A_HCD
A_ETD
B_HCD
B_ETD
cross−linker cleavage
A+XL_S
B+XL_L
A+XL_L
B+XL_S
intensity
mass
0
500000
1000000
1500000
500
1000
1500
z1
c1
z2
b2
c2
z2
z3
y3
z3
z4
y4
c2
c3
c3
c4
z4
y4
c4
c5
b5
z5
y5
LFKNLK+XL_S
LFKNLK+XL_L
HKQFWR+XL_S
HKQFWR+XL_L
